# The effects of the wenyang huoxue method on coronary heart disease heart failure: A protocol for systematic review

**DOI:** 10.1097/MD.0000000000019672

**Published:** 2020-04-03

**Authors:** Wenbo Han, Yong Zhao, Yahong Wang, Yanyan Dai, Jinhong Hao, Tianli Li, Xian Wang

**Affiliations:** a Dongzhimen Hospital, Affiliated Hospital of Beijing University of Chinese Medcine; b Institute of cardiovascular disease, Beijing University of Chinese Medicine, Beijing, China.

**Keywords:** Coronary heart disease, heart failure, protocol, systematic review, wenyang huoxue method

## Abstract

**Background:**

Coronary heart disease (CHD) has become the primary cause of heart failure (HF). Wenyang Huoxue method can significantly improve cardiac function in patients with CHD complicated with HF, but it has not been systematically evaluated for efficacy and safety.

**Methods:**

We will search China National Knowledge Infrastructure Database, Wanfang database, China Biomedical Literature Database, China Science Journal Database PubMed, Excerpt Medica Database, and Cochrane library. Clinical trial registrations, potential grey literature, related conference abstracts, and reference lists of identified studies will also be retrieved. The electronic database will be searched for literatures published from January 2000 to September 2019. Based on the heterogeneity test, data integration is performed using a fixed effect model or a random effects model. Changes in total effective rate in cardiac function will be assessed as primary outcome. 6-minute walk test, left ventricular ejection fraction, and plasma brain natriuretic peptide will be assessed as secondary outcomes. RevMan 5.3.5 will be used for meta-analysis.

**Results:**

This study will provide a high-quality comprehensive evaluation of the efficacy and safety of Wenyang Huoxue method for treating patients with CHD complicated with HF.

**Conclusions:**

This systematic review will determine whether Wenyang Huoxue method provides evidence for effective intervention in patients with CHD complicated with HF.

**Ethics and dissemination:**

This systematic review and meta-analysis of randomized controlled trials does not require ethical recognition, and the results of this paper will be published in an open access, internationally influential academic journal.

**Trial registration number:**

CRD42016025957

## 1 Introduction

Heart failure (HF) is the main presentation of the terminal stage of different cardiovascular diseases, defined as the inability of the heart to supply the peripheral tissues with the required amount of blood and oxygen to meet their metabolic demands.^[1]^ The growing burden of HF has been 1 of the most serious health problems facing society today, affecting an estimated 50 million people worldwide.^[2]^ While advances have been made in the treatment of HF, patients still have high morbidity.^[3]^ As we know, the most common cause of HF is ischemic heart disease due to impaired myocardial perfusion, mostly caused by an acute or chronic myocardial ischemia.^[4]^ Recent research also suggests that coronary events might be an important driver of death in HF with preserved ejection fraction.^[5]^ Coronary heart disease (CHD) complicated with HF has been a global problem. In China, the mortality rate of hospitalized HF patients is about 5.3%, and CHD has become 1 of the major complications of HF, accounting for 49.4%.^[6]^ The large number of patients with CHD and HF has brought great economic burden both to the country and its people.

The treatment of CHD complicated with HF has been constantly standardized. The application of medication such as angiotensin-converting enzyme inhibitors, β-blockers, aldosterone receptor antagonists and angiotensin-receptor neprilysin inhibitors have improved the clinical outcome of HF.^[7]^ Recently study shows that sodium glucose co-transporter 2 inhibitors reduce the risk of HF or death from cardiovascular disease, which was hailed a major victory in the fight against HF.^[8,9]^ However, the risks associated with sodium glucose co-transporter 2 inhibitors, such as amputation, limb ischemia and diabetic ketoacidosis, need to be taken seriously.^[10,11]^ On the other hand, although the CANTOS study has brought new hope for CHD, the risk of infection, high treatment costs and other aspects are not satisfactory, which will limit its clinical application.^[12,13]^ Patients with CHD and HF still face high mortality rate, low quality of life, and multiple side effects.^[3,14]^

Under the circumstance, traditional Chinese medicine (TCM), plays an irreplaceable role in the treatment of CHD complicated with HF. TCM treatment is widely used in the world for its good efficacy, safety, low cost and sustainable improvement of patients’ symptoms and prognosis. TCM believes that Yang deficiency and blood stasis is the common pathogenesis of CHD and HF, while wenyang huoxue method can be used to treat CHD complicated with HF, benefits from its functions of activating blood circulation, accelerating the clearance of blood oxygen free radicals and anti-inflammation.^[15–18]^ Although previous systematic reviews had shown good effects of TCM for treating HF patients, the quality of the studies has become a common concern. And there is still a lack of high-quality evidence for treating CHD complicated with HF patients with Wenyang Huoxue method. This present study was designed to perform a comprehensive systematic review and evaluation of the efficacy and safety of Wenyang Huoxue treatment for CHD complicated with HF.

## 2 Methods and analysis

### 2.1 Design and registration of the review

The protocol of this review has been registered with the international Prospective Register of Systematic Reviews (PROSPERO; registration number: CRD42016025957) and has been reported in accordance with the Preferred Reporting Items for Systematic Reviews and Meta-analyses guidelines.^[19]^

### 2.2 Inclusion criteria

#### 2.2.1 Type of study

Randomized controlled trials that using Wenyang Huoxue method combined with western medicine to treat CHD complicated with chronic HF patients, regardless of blinding, will be included in this study. Studies with a sample size smaller than 30 in the intervention group will be excluded.

#### 2.2.2 Types of participants

Patients with a confirmed clinical diagnosis of CHD complicated with HF using New York cardiology society's classification criteria for heart function will be included. There are no limitation in age, sex, nation, ethnicity, and disease stage.

#### 2.2.3 Types of interventions

The control group was treated with conventional treatments such as antiplatelet aggregation, statins, angiotensin-converting enzyme inhibitors, β-blockers, and aldosterone receptor antagonists. Patients in the treatment group were treated with the Wenyang Huoxue method and conventional treatments.

#### 2.2.4 Types of outcomes

The primary outcome will be the total effective rate in cardiac function, total effective rate = (total effective number)/total number × 100%. The secondary outcomes included the 6-minute walk test, left ventricular ejection fraction, and plasma brain natriuretic peptide.

### 2.3 Data sources and search methods

#### 2.3.1 Electronic searches

To identify all relevant studies, we systematically searched China National Knowledge Infrastructure Database, Wanfang database, China Biomedical Literature Database, China Science Journal Database, PubMed, Excerpt Medica Database, and Cochrane library, which was concluded from Jan 2000 to Sep 2019. The following search terms were used individualy or in combination: “coronary heart disease”, “heart failure”, “Wenyang Huoxue”, and “randomized controlled trial”. The search strategy for Medline will be searched via PubMed and is shown in Table 1.

**Table 1:**
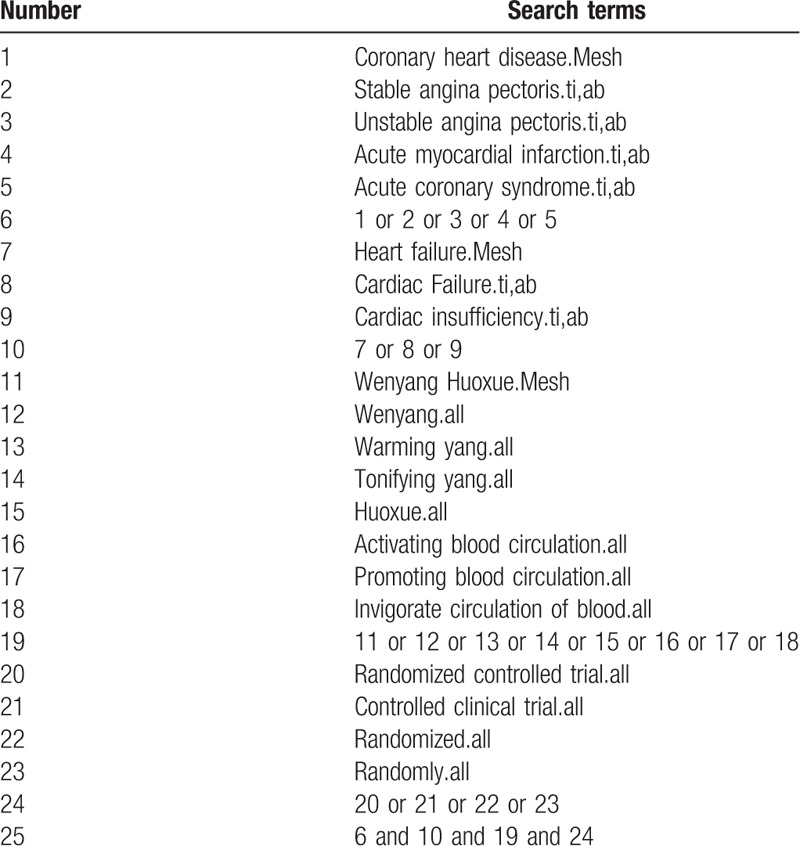
Search strategy used in Pubmed.

#### 2.3.2 Searching other resources

A reference list of research and systematic reviews will be reviewed and retrieved for additional testing. Potential gray literature will be searched in OpenGrey.eu. We will search for relevant conference abstracts to find eligible trials. In addition, we will search the World Health Organization International Clinical Trial Registration Platform and the clinical trial website ClinicalTrials.gov for all new comments related to this topic.

### 2.4 Data collection and management

**Figure 1:**
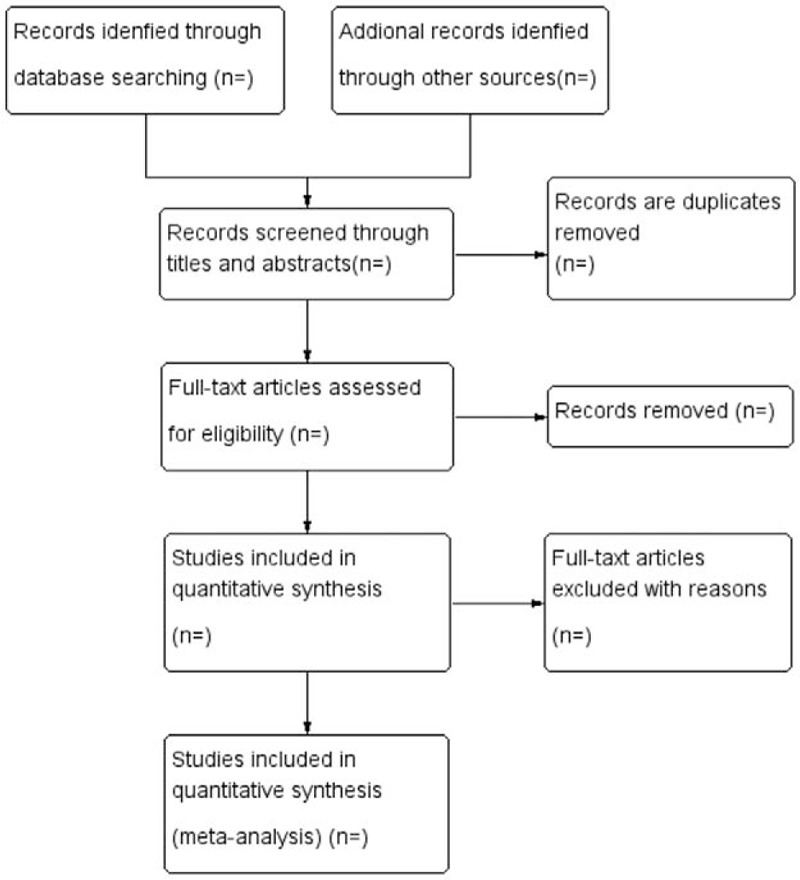
Flow diagram of the literature searching and study selection.

Based on the research criteria and search strategies identified above, 2 reviewers will review the topics and abstracts independently and use standardized data sheets to extract data including basic information such as patients, interventions, and outcomes. If the reviewers encountered inconsistencies, they would be resolved in a consultation with a third reviewer. See the PRISMA flow chart for the research selection (Fig. 1).

### 2.5 Data extraction and analysis

The RevMan 5.3.5 software provided by the Cochrane Collaboration was used for data analysis. Dichotomous data are expressed as relative risk, continuous outcomes are presented as weighted mean difference or standerd mean difference, and the 95% confidence intervals were calculated for both. The meta analysis was performed in the intervention and control groups.

### 2.6 Assessment of risk of bias in the included studies

To ensure that the selected literature was of high quality, we used the RevMan 5.3.5 to assess the studies systematically and comprehensively, according to the 7-parameter set. These were sequence generation, allocation concealment, blinding of participants, and personnel, blinding of outcome assessors, incomplete outcome data, selective outcome reporting, and other sources of bias. Each item will be rated as high risk bias, low risk bias, and uncertain bias. When there is any disagreement, it will be resolved through discussion or consultation with the third reviewer.

### 2.7 Assessment of heterogeneity

The statistical heterogeneity was considered significant if the *I*^2^ index exceeded 50% or *P* < .1. In the absence of significant heterogeneity, we pooled the data using fixed (*I*^2^ < 50%) or random (*I*^2^ > 50%) effects models.

### 2.8 Assessment of reporting biases

When more than 10 studies are included, a funnel plot will be used to detect report bias.

### 2.9 Subgroup analysis and sensitivity analysis

We will select subgroup analysis and sensitivity analysis to detect sources of possible clinical or methodologic heterogeneity.

### 2.10 Grading the quality of evidence

The GRADE profiler software (Version 3.6, The GRADE Working Group) will be used to analyze the quality level of evidence.

## 3 Discussion

HF has been a global problem, which is associated with high mortality and is estimated to have cost about US$100 billion in 2012.^[20,21]^ The main risk factors of HF is coronary artery disease (CAD).^[4]^ Although the incidence of acute myocardial infarction has decreased worldwide, the prevalence of ischaemic HF has increased.^[22]^ The treatment of CHD complicated with HF has important clinical significance to reduce the economic burden and improve the quality of life. However, we are unable to do much more than reduce congestion with diuretics in HF patients.^[23,24]^ Now TCM gives us a new option. Researchers have developed a large number of clinical studies in Wenyang Huoxue method of CHD complicated with HF patients. Nevertheless, no systematic review related to Wenyang Huoxue method for CHD complicated with HF has been published in English so far. In this paper, we present a protocol for a systematic review of Wenyang Huoxue method for CHD complicated with HF patients. We hope this review will facilitate clinicians when making decisions.

## Author contributions

**Conceptualization:** Wenbo Han, Yong Zhao, Xian Wang.

**Data curation:** Wenbo Han, Yanyan Dai, Jinhong Hao.

**Formal analysis:** Wenbo Han, Yong Zhao, Tianli Li.

**Project administration:** Wenbo Han, Yong Zhao.

**Supervision:** Xian Wang, Yahong Wang.

**Writing – original draft:** Wenbo Han.

**Writing – review & editing:** Wenbo Han, Yong Zhao.
